# Substrate Compositional Variation with Tissue/Region and *Gba1* Mutations in Mouse Models–Implications for Gaucher Disease

**DOI:** 10.1371/journal.pone.0057560

**Published:** 2013-03-08

**Authors:** Ying Sun, Wujuan Zhang, You-Hai Xu, Brian Quinn, Nupur Dasgupta, Benjamin Liou, Kenneth D. R. Setchell, Gregory A. Grabowski

**Affiliations:** 1 Division of Human Genetics, University of Cincinnati College of Medicine, Cincinnati, Ohio, United States of America; 2 Division of Pathology and Laboratory Medicine, University of Cincinnati College of Medicine, Cincinnati, Ohio, United States of America; 3 Cincinnati Children’s Hospital Medical Center and the Department of Pediatrics, University of Cincinnati College of Medicine, Cincinnati, Ohio, United States of America; MUSC SC College of Pharmacy, United States of America

## Abstract

Gaucher disease results from *GBA1* mutations that lead to defective acid β-glucosidase (GCase) mediated cleavage of glucosylceramide (GC) and glucosylsphingosine as well as heterogeneous manifestations in the viscera and CNS. The mutation, tissue, and age-dependent accumulations of different GC species were characterized in mice with *Gba1* missense mutations alone or in combination with isolated saposin C deficiency (C*). *Gba1* heteroallelism for D409V and null alleles (9V/null) led to GC excesses primarily in the visceral tissues with preferential accumulations of lung GC24∶0, but not in liver, spleen, or brain. Age-dependent increases of different GC species were observed. The combined saposin C deficiency (C*) with V394L homozygosity (4L;C*) showed major GC18∶0 degradation defects in the brain, whereas the analogous mice with D409H homozygosity and C* (9H;C*) led to all GC species accumulating in visceral tissues. Glucosylsphingosine was poorly degraded in brain by V394L and D409H GCases and in visceral tissues by D409V GCase. The neonatal lethal N370S/N370S genotype had insignificant substrate accumulations in any tissue. These results demonstrate age, organ, and mutation-specific quantitative differences in GC species and glucosylsphingosine accumulations that can have influence in the tissue/regional expression of Gaucher disease phenotypes.

## Introduction

Gaucher disease is an autosomal recessively inherited disorder caused by mutations in *GBA1* that encodes lysosomal acid β-glucosidase (GCase) (E.C. 3.2.1.45). Defective GCase activities lead to tissue accumulations of the substrates, glucosylceramide (GC) and glucosylsphingosine [1]. Three types of Gaucher disease are classified by their phenotypic manifestations: Type 1 has primarily hepatomegaly, splenomegaly, hematological, and bone disease with great variability in disease expressivity [1]. Types 2 and 3 are neuronopathic variants that are distinguished by the presence and degree of neuronopathic disease. Type 2 patients have more severe, rapidly progressive CNS deterioration, whereas type 3 patients have more variable severity and progression of visceral and CNS involvement [1]. Over 350 mutations in *GBA1* have been reported worldwide in Gaucher disease patients [2], [3]. Although imperfect, there are reasonable correlations between the phenotype and level of mutant residual GCase activity [1]. For example, N370S GCase is associated with Gaucher disease type 1 and has greater intrinsic enzyme activity than L444P or D409H GCases, which are associated with neuronopathic variants [4]. The V394L allele is usually in a heteroallelic state, e.g. N370S/V394L with mild disease, or L444P/V394L with CNS and visceral involvement [5], [6]. However, the understanding of the heterogeneity of differential visceral organ and/or CNS regional involvement remains elusive.

Two GCase substrates, GC and glucosylsphingosine, accumulate in visceral organs and CNS regions; GC shows the greatest accumulation by mass. GC is composed of β-D-glucose and ceramide. The latter contains sphingosyl as well as fatty acid acyl chains (FAAC) of varying chain length from 16 to >26 carbons [7]. The fatty acid acyl composition analyses of GC from human visceral tissues [7], [8] showed that GC16∶0, GC22∶0 and GC24∶0 are the major species, and GC18∶0 is the most abundant GC in CNS [7], [9], [10]. In spleens from Gaucher disease types 1 and 3 patients, the longer chain species, GC22∶0 and GC24∶0 have the greatest increased levels [7], [11]. Glucosylsphingosine is the deacylated form of GC and belongs to the lyso-glycosphingolipid family [12]. In healthy individuals, glucosylsphingosine is almost undetectable in tissues, but is variably elevated in Gaucher disease variant spleens and livers [13]–[15]. Glucosylsphingosine is toxic to cultured neurons when added to the media [16], and is markedly elevated in CNS regions from Gaucher disease types 2 and 3 patients [10], [15], [17].

To elucidate potential relationships between different mutant GCases and the GC species/glucosylsphingosine, the age- and tissue-dependent accumulations of these substrates were determined in several *Gba1* mutant mouse models. These results provide insight into the regional and tissue specific variation of GC species and glucosylsphingosine accumulations in Gaucher disease mice, and provide a basis for comparative human studies.

## Materials and Methods

### Materials

The following were from commercial sources**:** Synthetic sphingolipid standards including glucosylsphingosine, N-acyl glucosylceramide (C8, C12, C16, C18, and C24∶1) in 99% purity (Avanti Lipids, Inc, Alabaster, AL). Supelcosil-LC-18-DB column, Supelco 2.1*250 mm column, ammonium formate, formic acid, methanol and chloroform (Sigma-Aldrich, Corp., St. Louis, MO). 4-methylumbelliferyl-β-D-glucopyranoside (4MU-Glc) (Biosynth AG, Switzerland). Conduritol B epoxide (CBE) and sodium taurocholate (Calbiochem, La Jolla, CA). Sephadex™ G-25 Fine column (GE Healthcare Bio-Sciences AB, Pittsburgh, PA).

### *Gba1* Mutant Mice and Tissue Collection

*Gba1* mutant mice were generated as described [18]. The 9V/null mice were created by back-crossing D409V/D409V with *Gba1* null/WT mice [18]. The mouse models with combined *Gba1* mutations and saposin C deficiency were generated by back-crossing of saposin C deficient mice (C−/−, or C*) with specific *Gba1* mutant mice [19]. The resultant mice were analogous to human mutations for C−/− and the missense *Gba1* mutants; D409H/D409H = 9H/9H, V394L/V394L = 4L/4L, D409V/D409V = 9V/9V and N370S/N370S = 0S/0S. The *Gba1* homozygous mutants with C* were designated 9H;C*, 4L;C*, 9V;C*, and 0S;C*. The strain backgrounds for WT control mice were C57BL/129 and FVB. The mice were maintained in microisolators in accordance with institutional guidelines under Cincinnati Children’s Hospital Research Foundation Institutional Animal Care and Use Committee (IACUC) approval at Cincinnati Children’s Hospital Research Foundation. The tissues were collected from the mice after perfusion with saline and stored at −80°C.

### GCase Activity Assays

Mouse tissues were homogenized and assayed in 0.25% sodium taurocholate and 0.25% Triton X-100. GCase activities were determined fluorometrically with 4MU-Glc in the presence and absence of CBE (1 mM) using a SpectraMax M5e (Molecular Devices). Tissue GCase activities were analyzed in parallel with WT control as described [18].

### Glycosphingolipid Analyses

Extractions of glycosphingolipids were as described [20]. Following methanol/chloroform/water (2∶1:0.6, v/v/v) extraction, the extracts from frozen tissues were subjected to KOH alkaline methanolysis (0.3 N, 0.6 mL) at 37°C (1h) to remove potentially interfering glycolipids. After cooling, approximately 50 µL of 3N HCl was added to neutralize the solution, followed by water (50 µL) and chloroform (1.2 mL). To remove non-lipid contaminants, the samples were brought to 5 mL with chloroform:methanol:water (60/30/4.5, v/v/v) and loaded onto a Sephadex G-25 Fine column equilibrated in the above solvent. The column was washed with 5 mL of chloroform:methanol (2∶1, v/v). Sphingolipids were collected (10 mL) and dried under nitrogen gas.

Analyses of GC and glucosylsphingosine were carried out by LC-ESI-MS/MS with minor modifications [20]. Online chromatographic separation for GC and glucosylsphingosine was achieved using a Supelcosil-LC-18-DB column. Gradient elution with a mobile phase of methanol and water charged with ammonium formate and formic acid was employed as described for the separation of sphingolipid species of varying acyl chain length. GC in brain was resolved from galactosylceramide by hydrophobic interaction liquid chromatography (HILC) and quantified by MS. The absence, i.e., undetectable, of galactosylceramide was verified in the visceral tissues (liver, lung and spleen) by HILC-MS/MS. Optimized parameters for GC and glucosylsphingosine were determined with individual standard compounds (Matreya, LLC and Avanti Polar lipids, Inc.). Calibration curves were prepared for GC16, GC18, and GC24∶1 using GC12 as the internal standard. Quantification of GC species with various fatty acid chain lengths was established from the calibrations curve of each species with closest number of chain length. The quantification of glucosylsphingosine was based on the calibration curve using C8-β-glucosylceramide as internal standard. The linear response for GCs and glucosylsphingosine was 50 pg–25 ng. The extracted tissues samples were suspended in methanol containing the internal standard and 10 µL was injected for LC-ESI-MS/MS measurements.

The GC species and glucosylsphingosine levels were normalized to tissue weight (mg). Age-matched WT tissues were analyzed in parallel with mutant mouse samples. The data were analyzed by Student’s t-test using GraphPad Prism 5 software. The preference of GC species or glucosylsphingosine accumulation for each mutant was analyzed by Tukey’s HSD (honestly significant difference) test to compare means of the proportion GC species in each mutant and WT tissue. The proportion of each GC species was determined using the total of 6 GC species (GC16∶0, GC18∶0, GC20∶0, GC22∶0, GC24∶0 and GC24∶1). Glucosylsphingosine concentrations were used directly for the analyses. The Tukey's HSD test was performed and plot was generated using JMP software (SAS Institute Inc., Cary, North Carolina). The mutant mice, 9H/9H, 4L/4L, 9V/9V, 9V/null and 9H;C* at 52 wks and 4L;C* at 6 wks, and WT mice at 6 wks and 52 wks were included in the analyses. The value P<0.05 indicates a significant difference between WT and mutant mice.

### Heat Map Construction

The levels of GC species and glucosylsphingosine in the heat map are displayed as the fold-change obtained for each tissue from mutant mice relative to the average value of age-matched WT. A global comparison of the different GC species and glucosylsphingosine in a heat map was performed using Gene Spring 11.5.1 Version (Agilent Technologies, Santa Clara, CA). The fold-change values of triplicate or quadruplicates animals for most of the conditions in the four *Gba1* mutant mouse models (95 samples) were imported into a single experiment and a heat map was generated. The fold-change value is mapped to a color-intensity value and depicted by a color gradient bar. Yellow indicated a fold- change value of one. Color gradient from yellow to red referred to the fold-change value is >1 and from yellow to green referred to the fold change value is <1.

## Results

### Gaucher Disease Mouse Models and Their Phenotypes

Two groups of *Gba1* mutant mice were evaluated for substrate levels in viscera and brain (Table 1). In the first group, mice were homozygous for knock-in *Gba1* point mutations, including those encoding 4L/4L, 9H/9H, and 9V/9V, and 9V/null [18]. The GCase activities by *in vitro* assay in all these mice range from ∼2–11% WT level in visceral tissues and ∼21–27% in the CNS (Table 1) [18]. All of these *Gba1* point mutant mice have normal life spans. Because the instability of the D409V GCase protein and activity [4], the degrees of reduced *in vitro* activity in 9V/null extracts could not be precisely determined and were somewhat variable. In 9V/null mice, storage cells (macrophages) develop in the lung and liver by 3 mos. In 4L/4L, 9H/9H, and 9V/9V mice, a few storage cells are present in the spleen by 7 mos. These mutant mice have less visceral storage than Gaucher disease type 1 patients. As described, 0S/0S mice had a neonatal lethal phenotype due to a skin permeability defect [18]. Human patients with N370S homozygosity do not display this skin defect.

**Table 1:** Gaucher disease mouse models.

Mouse	Genotype		Strain background	Life span	GCase Activity^a^ (%WT)					Phenotypes
*Gba1 mutation mice*					Liver	Lung	Spleen	Brain	Age^d^	
9H/9H		D409H/D409H	C57BL/129Sv	>2 years	5.4±2.4	6.7±0.8	9.3±2.2	27.8±6.8	7.5 mos	Few visceral storage cells
4L/4L		V394L/V394L	C57BL/129Sv	>2 years	4.0±1.4	7.0±1.4	10.9±2.7	27.4±3.6	13 mos	Few visceral storage cells
9V/9V		D409V/D409V	C57BL/129Sv	>2 years	2.5±0.9	5.4±2.2	6.2±2.7	22.5±3.8	7.5 mos	Few visceral storage cells
9V/null		D409V/null	C57BL/129Sv/FVB	>2 years	3.9±0.4	4.0±1.3	6.8±3.2	21.4±1.6	6.5 mos	Visceral storage cells
0S/0S		N370S/N370S	C57BL/129Sv	<1 day (Lethal)	13.5±3.7	30.3±2.2	nd c	17.4±1.0	<1 day	Skin permeability defect
Combined Gba1 mutation and saposin C−/− (C*) mice										
9H;C*		D409H/D409H; C−/−	C57BL/129Sv	>1 year	4.6±0.6	7.5±1.2	7.4±0.2	31.6±3.0	3 mos	Visceral storage cells and delayed neuropathic defect
4L;C*		V394L/V394L; C−/−	C57BL/129Sv	∼48 days	2.6±0.6	4.2±1.1	6.8±1.0	10.0±1.3	6 wks	Neuropathic defect
9V;C*		D409V/D409V; C−/−	C57BL/129Sv	<1 day (Lethal)	3.5±1.3 (7.5±1.1)^b^	6.9±4.9 (14.2±4.8)^b^	nd	16.0±5.4 (31.3±12.3)^b^	<1 day	Skin permeability defect
0S;C*		N370S/N370S; C−/−	C57BL/129Sv	<1 day (Lethal)	6.4±1.2	14.9±3.0	nd	12.5±0.7	<1 day	Skin permeability defect

a GCase activities in 9H/9H, 4L/4L, 9V/9V and 9V/null mice [18] and in 4L;C* mice [23] were determined previously (n = 3–4 mice).

b GCase activities in age-matched 9V/9V mice at 1 day of age were in parenthesis.

c nd, not determined.

d GCase activities were assayed from the mice at indicated age.

The second group was derived from mice with *Gba1* point mutations in combination with isolated saposin C deficiency (WT;C−/− or WT;C* where WT is the wild type *Gba1*). The rationale for generating combined mutants is based on saposin C’s protease protective effect for normal and mutant GCases [21]. In the context of C*, WT GCases have ∼50% decreased protein and *in vitro* activities in mouse tissues. Importantly, WT;C* mice do not develop CNS disease until after 12–15 mos. and have no significantly increased GC and glucosylsphingosine accumulations [19], [22]. Backcrossing of the *Gba1* point mutants (pm), 4L/4L, 9H/9H, 9V/9V and 0S/WT with C* mice, led to mice that are designated pm;C*, e.g., 4L/4L;C* is 4L;C*. These pm;C* mice had variety of different phenotypes (Table 1). Differences of *in vitro* activity could not be reproducibly differentiate between 9H/9H mice and 9H;C* (Table 1); this is likely due to the instability of D409H GCase protein [4]. The 9H;C* mice had visceral involvement with large numbers of storage cells in the liver and fewer in the spleen and lung (data not shown). The 9H;C* mice developed CNS involvement and histopathology at ∼1 year, which was earlier than WT;C* mice [19]. In comparison, the 4L;C* mice had early onset (∼30 days) of neuronopathic disease, a shorten life span, ∼48 days, and nearly normal visceral histopathology [23]. GCase activities in 4L;C* mice were ∼50% of those in 4L/4L mice (Table 1). Two variants, 9V;C* and 0S;C*, had pre- or neo-natal lethal phenotypes with death within 24 hrs after birth (Table 1). The skins of 9V;C* and 0S;C* pups were wrinkled and dry, similar to the 0S/0S and *Gba1* null/null mice, that is indicative of a severe disruption of the skin permeability barrier [18], [24]. GCase activities in 9V;C* were ∼50% of 9V/9V levels and in 0S;C* were ∼50% of 0S/0S levels in the liver, lung, and brain (Table 1).

**Figure 1:**
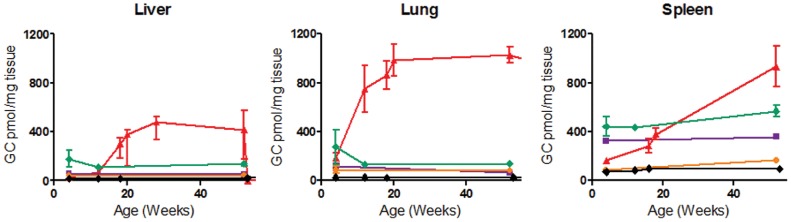
Glucosylceramide (GC) in *Gba1* mouse tissues. Total GC levels in liver, lung, and spleen from 9V/null (red), 9H/9H (violet), 9V/9V (green), and 4L/4L (orange) mice. In 9V/null the GC levels progressively accumulated from 4 to 52 wks and at greater rates in the lung than in liver and spleen. 9H/9H, 9V/9V, and 4L/4L mice had GC increases slightly above WT (black) levels. Data are mean±S.E. (n = 3–4 mice).

The mouse tissues used in lipid analyses were collected from *Gba1* mutant and WT mice. All the mutants were in the C57BL/129Sv background, except the 9V/null mice were in the C57BL/129Sv/FVB background (Table 1). Because the mixed background of the mutant mice, WT tissues from two different backgrounds, FVB strain and C57B/129 mixed strain, were included in the analyses. For <1d and 6 wk mutants in the C57B/129Sv background, strain matched C57B/129Sv WT were used. For age dependent GC level changes shown in Fig 1, FVB WT mice were used. For 52 wk mutant mice, FVB and C57B/129Sv WT were used. The differences between the variant WT GC levels in the different strains were within the variation range of the assays.

### GC Levels in Visceral Tissues of *Gba1* Mutant Mice

Total GC concentrations in the visceral tissues from 9H/9H, 4L/4L, and 9V/9V mice were 2- to 6-fold increased relative to WT (Fig.1) and the GC concentrations were relatively stable over time. In comparison, GC concentrations in the viscera from 9V/null mice were much greater, increased rapidly after birth, and plateaued by 20 wks in livers and lungs; a continued increase in GC concentration was observed in spleen. GC concentrations were much greater in lung (38-fold) than in liver (16-fold) and spleen (10-fold) of these mice compared to WT. The GC concentrations in the brain of all the *Gba1* pm mice were <2-fold above and paralleled WT levels from 4 to 52 wks (data not shown).

### Tissue Variation in GC Species in *Gba1* Mutant Mice

**Figure 2:**
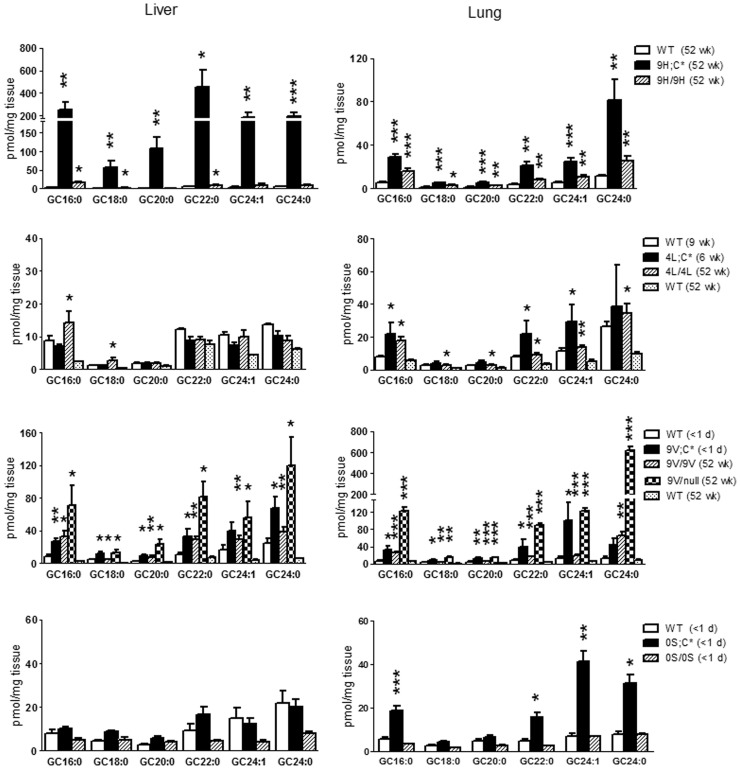
GC species in the liver and lung. Each panel presents GC species levels in mice having the indicated *Gba1* mutation: D409H (9H/9H and 9H;C*), V394L (4L/4L and 4L;C*), D409V (9V/9V, 9V/null, and 9V;C*) and N370S (0S/0S and 0S;C*). The panels are grouped to reflect the various mutations to facilitate comparison of the GC levels. The ordinates differ to facilitate visualizing the differing levels of GC. Age-matched WT tissues were controls for each analysis. 4L;C* lung at 6 wks was compared to WT at 9 wks. Each GC species level in the mutant was analyzed relative to age-matched WT using Student’s t-test. *, p<0.05, **, p<0.01, ***, p<0.0001, ****, p<0.00001. The results and error bars are the mean+S.E. (n = 3–4 mice).

**Figure 3:**
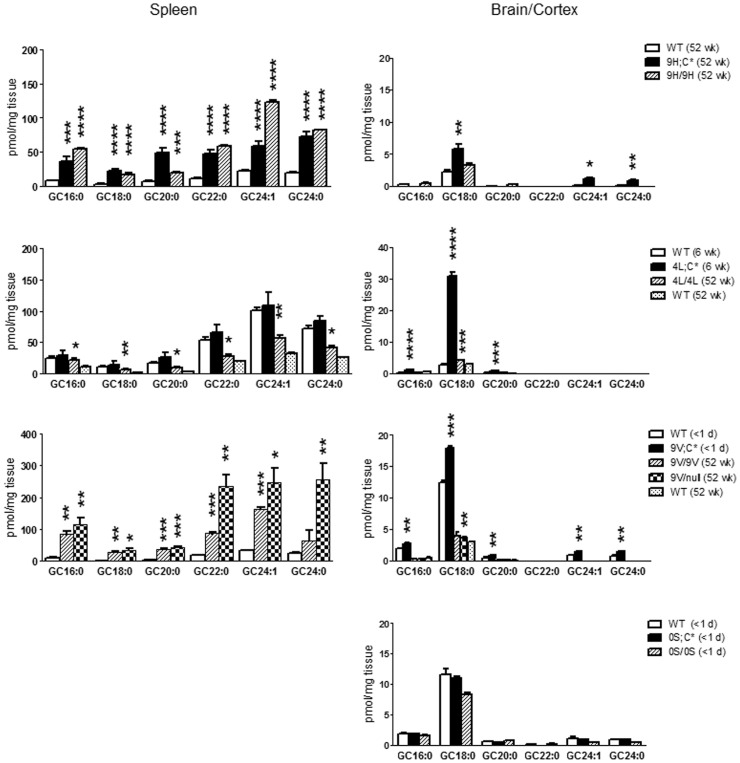
GC species in the spleen and brain. GC species in the spleen (9H/9H, 9H;C*, 4L/4L, 4L;C*, 9V/9V, and 9V/null), whole brain (9H;C*, 4L;C*, 9V;C*, 0S/0S, and 0S;C*) or cerebral cortex (9H/9H, 9V/9V, 4L/4L, and 9V/null) are presented. GCs in the brain extracts were resolved from galactosylceramide by hydrophobic interaction liquid chromatography and quantified by MS. The spleen of ∼1 day-old mice (0S/0S, 9V;C* and 0S;C*) were not determined. 4L;C* spleen at 6 wks was compared to WT at 9 wks. The data analyses were as in Figure 2.

GC species were profiled in liver, lung, and spleen tissues from *Gba1* mutant and WT mice (Figs. 2 and 3, Table 2). The abundant GC species in visceral tissues were GC16∶0, GC22∶0, GC24∶1 and GC24∶0. In comparison, in CNS the GC18∶0 was predominant. The minor amounts of GC18∶1, GC20∶1, GC22∶1, GC26∶0 and GC26∶1 (<1% of total) are not presented on the graphs. GC concentrations were at WT levels in CNS from heterozygotes for each of the *Gba1* pm mice (data not shown).

**Table 2:** Fold changes of GC species levels in *Gba1* mutant mice tissues relative to WT.

Tissue	GC species		Genotypes						
			9H/9H	9H;C*	4L/4L	4L;C*	9V/9V	9V/null	9V;C*
		Age	52 wks	52 wks	52 wks	6 wks	52 wks	52 wks	1 day
Lung	GC16∶0		2.9^#^ a	5.3 *^#^*	3.3 ^#^	2.6 ^#^	4.8 ^#^	22.5 ^#^	5.0 ^#^
	GC18∶0		2.7 ^#^	3.8 ^#^	2.9 ^#^	1.3	4.7 ^#^	14.9^#^	2.8
	GC20∶0		2.2 ^#^	4.0 ^#^	2.5 ^#^	1.8	4.8 ^#^	11.8 ^#^	2.6
	GC22∶0		2.3 ^#^	5.6 ^#^	2.7 ^#^	2.8 ^#^	5.1 ^#^	27.6 ^#^	4.8
	GC24∶1		2.0 ^#^	4.6 ^#^	2.6 ^#^	2.5 ^#^	3.9 ^#^	22.8 ^#^	8.1 ^#^
	GC24∶0		2.7 ^#^	7.4 ^#^	3.7 ^#^	1.5	6.8 ^#^	64.9 ^#^	2.9
Liver	GC16∶0		6.9 ^#^	66.2 ^#^	6.2 ^#^	0.8	9.4 ^#^	31.1 ^#^	2.7 ^#^
	GC18∶0		6.9 ^#^	69.1^#^	7.4 ^#^	1.0	8.5 ^#^	35.4 ^#^	2.1
	GC20∶0		1.6	82.8 ^#^	1.6	1.0	5.2 ^#^	21.1 ^#^	2.4
	GC22∶0		1.3 ^#^	63.1 ^#^	1.2	0.7	2.7 ^#^	10.5 ^#^	2.9 ^#^
	GC24∶1		2.3	35.2 ^#^	2.2	0.7	4.7 ^#^	12.7 ^#^	2.2 ^#^
	GC24∶0		1.6	30.6 ^#^	1.4	0.8	4.3 ^#^	19.3 ^#^	2.6 ^#^
Spleen	GC16∶0		4.9 ^#^	6.3 ^#^	1.9 ^#^	1.2	7.5^#^	10.3 ^#^	nd c
	GC18∶0		8.6 ^#^	6.8 ^#^	3.6 ^#^	1.3	14.1^#^	16.7 ^#^	nd
	GC20∶0		5.4 ^#^	6.7 ^#^	2.4 ^#^	1.5	9.8^#^	11.4 ^#^	nd
	GC22∶0		3.0 ^#^	7.0 ^#^	1.4 ^#^	1.2	4.4^#^	12.0 ^#^	nd
	GC24∶1		3.7 ^#^	3.6 ^#^	1.7 ^#^	1.1	5.0^ #^	7.5 ^#^	nd
	GC24∶0		3.2 ^#^	5.5 ^#^	1.6 ^#^	1.2	6.4	10.0 ^#^	nd
Brain	GC16∶0		2.0	<LQD b	0.8	2.7 ^#^	0.8	0.8	1.4 ^#^
	GC18∶0		1.5	2.6 ^#^	1.4 ^#^	10.6 ^#^	1.3	1.2 ^#^	1.4 ^#^
	GC20∶0		3.0	<LQD	3.0	5.0 ^#^	3.0	2.5	1.7 ^#^
	GC22∶0		<LQD	<LQD	<LQD	<LQD	<LQD	<LQD	<LQD
	GC24∶1		<LQD	4.0 ^#^	<LQD	<LQD	<LQD	<LQD	1.6 ^#^
	GC24∶0		<LQD	3.2 ^#^	<LQD	<LQD	<LQD	<LQD	1.7 ^#^

a # indicates significantly changed GC species level relative to WT. Data present mean of 3–4 mice per genotype.

b <LQD refers to lower than quantitative detection limit.

c nd, not determined.

The GC species tissue concentrations in the 9H/9H and 9H;C* mice were compared. In 9H/9H mouse lung at 52 wks, all GC species were significantly increased relative to WT, whereas in liver only GC16∶0, GC18∶0, and GC22∶0 were elevated (Fig. 2, Table 2). 9H;C* mice had massively increased (30- to 80-fold) GC species in liver and less so in lung (4- to 7-fold) (Table 2). In spleen, the GC species were increased similarly (3- to 9- fold) in both genotypes. GC18∶0, the major GC species in the brain, was increased in 9H;C*, but not in 9H/9H brain (Fig. 3). Although at low levels, GC24∶1 and GC24∶0 also were increased in 9H;C* brain.

GC species accumulations were evaluated in 4L/4L and 4L;C* mice. In 4L/4L at 52 wks, GC16∶0 and GC18∶0 showed minor elevations in liver (Fig. 2), whereas most GC species were increased in lung (Fig. 2). By 6 wks, In the lungs and livers of 4L;C* mice, the GC species were at very similar levels to those in age-matched WT (6–9 wk). In the spleen, GC species were increased in 4L/4L at 52 wks, but not in 4L;C* mice at 6 wks (Fig. 3). GC18∶0 was significantly increased in 4L/4L cerebral cortex and 4L;C* brain compared to their respective age-matched control. Also, relative to 4L/4L mice at 52 wks, the brain of 4L;C* mice had 7.6-fold greater GC18∶0 (Fig. 3 and Table 2). This finding demonstrates that a V394L mutation has significant effects on brain GC degradation.

GC species were evaluated in three models that contained the D409V mutation, i.e., 9V/9V, 9V/null, and 9V;C*. Compared to the respective age-matched WT, most of the GC species were elevated in lung, liver, and spleen. In these organs, most GC species were 2- to 10-fold greater in 9V/null compared to 9V/9V (Figs. 2 and 3, Table 2). In lungs and livers, the GC24∶0 and GC16∶0, respectively, showed the greatest increases, whereas in spleen the GC22∶0, GC24∶1, and GC24∶0 showed the largest increases with the 9V/null being consistently greater. The 9V;C* mice died at <1 day, but all GC species already were increased in liver and lung. Spleen of the pups was of insufficient size to provide reliable measurements. In brain, GC18∶0 levels were not increased in 9V/null or 9V/9V cortex. However, brain of 9V;C* (<1 day) showed increases in GC18∶0, GC16∶0, GC24∶1 and GC24∶0 (Fig. 3). These analyses revealed that the D409V GCase led to GC accumulations in both visceral tissues and brain with greater accumulations of GC24∶0 in the lung and GC16∶0 in the liver.

Substrate levels were evaluated in newborn 0S/0S and 0S;C* pups. These mice survived for ∼1 day. They succumbed to a skin permeability defect similar to that in *Gba1* null/null mice. 0S;C* mice showed significant accumulations of most GCs in the lung, but not in the liver and brain (Figs. 2 and 3). Spleen lipids could not be analyzed in these mice due to inadequate tissues. Thus, the N370S GCase did not lead to large early GC accumulations and the 0S;C* showed lesser accumulations than the 9V;C*.

Brain galactosylceramides that contain more long chain FAAC species were analyzed and were not altered in these mutants compared to WT (data not shown).

### Glucosylsphingosine Levels in *Gba1* Mutant Mice

**Figure 4:**
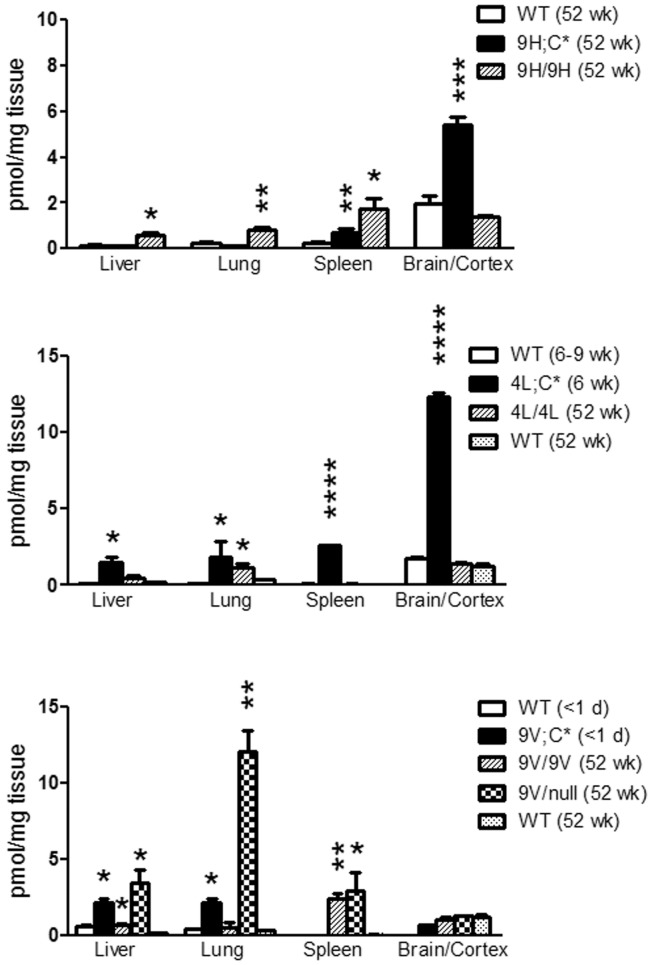
Glucosylsphingosine levels in *Gba1* mutant mice. Each graph depicts glucosylsphingosine levels in four tissues from mice having the same *Gba 1* mutation: D409H (9H/9H and 9H;C*), V394L (4L/4L and 4L;C*), D409V (9V/9V, 9V/null and 9V;C*) and N370S (0S/0S and 0S;C*). Age-matched WT tissues were included as control. The data analyses were as in Figure 2.

In the 9H/9H-based mice, glucosylsphingosine concentrations were increased in 9H/9H visceral tissues to a greater extent than in 9H;C*. This substrate was increased (2.8-fold) only in the cerebral cortices/brains of 9H;C* mice (Fig. 4). In comparison, in 4L/4L-based models, glucosylsphingosine concentrations in visceral tissues from 4L;C* mice were greater than those in 4L/4L. Also, very large increases (∼5.1-fold) were found in 4L;C* brain. In 9V/9V-based mice, glucosylsphingosine accumulated in the visceral tissues, but not in brain or isolated cerebral cortex (Fig. 4). Significant amounts of glucosylsphingosine were present in 9V/null lungs (Fig. 4). Slightly elevated glucosylsphingosine concentrations were detected in the liver and lung of 9V;C* and 9V/9V, and the spleen of 9V/9V and 9V/null mice (Fig. 4). 0S/0S and 0S;C* mice had no detectable change in glucosylsphingosine concentrations compared to age-matched WT (data not shown). These results indicate that glucosylsphingosine was poorly degraded by V394L and D409H GCases in the brain and D409V GCase in the visceral tissues.

### Mutation Associated Preferential GC Species and Glucosylsphingosine Accumulation

**Figure 5:**
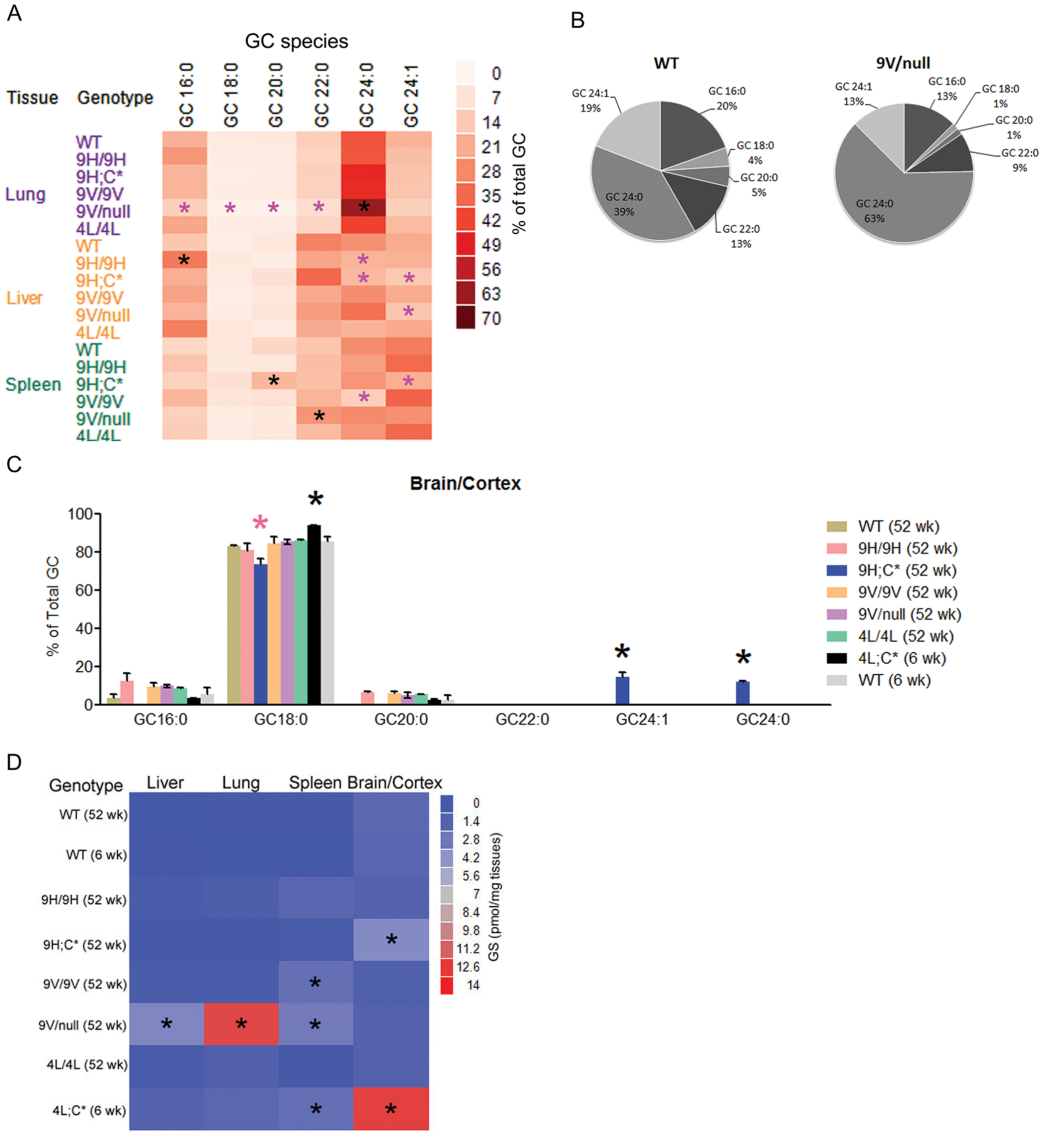
Analyses of substrate accumulation in *Gba 1* mutant mice. (A) The mean proportion of each GC species relative to total GC (6 GC species) in age-matched *Gba 1* mutants and WT (52 wks) visceral tissues were plotted. In the lung, GC24∶0 was significantly increased in 9V/null, whereas other species were reduced. In the liver, proportion of GC16∶0 was significantly increased in 9H/9H, but not in liver from other mutant mice. GC24∶0 had reduced proportions in 9H/9H and 9H;C* livers, whereas GC24∶1 was increased in 9V/null and 9H;C* livers compared to WT and other mutants. In the spleen, the proportion of GC20∶0 was significantly increased in 9H;C*. Reduced GC species were present in spleen of some mutant mice: GC24∶0 in 9H;C*, GC20∶0 in 9V/null, and GC24∶0 in 9V/9V. The black stars indicate that the proportion of a GC species was significantly increased and the pink stars indicate that the proportion of a GC species was reduced relative to WT. The color gradients indicate the proportion of total GC species represented by GCs with a defined FAAC. (B) The pie charts show the proportions of each GC species in 9V/null lung and WT at 52 wks. The GC24∶0 was 63% in 9V/null lung compared with 39% in WT. The proportions of the other GC species were concomitantly reduced in 9V/null lung. (C) In brain extracts, the proportion of GC18∶0 was significantly increased only in 4L;C* (black star) mice. In 9H;C* brain GC18∶0 was reduced (pink star) and long chain GC species, GC24∶0 and GC24∶1, were significantly increased (black stars). The proportion of each GC species in WT brain at 6 wks and 52 wks were included in the analyses. (D) Glusosylsphingosine concentrations in tissues from each mutant were plotted as the mean of concentration. Significant increases of glusosylsphingosine (black stars) were in 4L;C* brain and spleen, 9H;C* brain, and 9V/null lung, liver and spleen. The glucosylsphingosine levels in WT tissues at 6 wks and 52 wks were included in the analyses. The color gradient indicates glucosylsphingosine concentrations (pmol/mg tissues). The data in (A), (C) and (D) were analyzed with Tukey’s HSD test. The samples that showed significant difference from WT and other mutants (p<0.05) were marked with star (*).

To analyze GC species accumulations for each mutant mouse variant, the species concentrations were converted to the proportion of each GC species relative to total GC (6 major species). The differences of species proportions were compared between mutant to mutant and mutant to age-matched WT using the one-way Tukey’s HSD test. In visceral tissues, the proportion of long chain GC24∶0 was significantly increased in 9V/null lung compared to WT and other mutants (Fig. 5A). Total GC was massively increased in the lung of 9V/null mice and the proportion of GC24∶0 at 52 wks was 2.2-fold >WT. This proportion increased progressively from 4 wks (data not shown). The proportions of other GC species in 9V/null lung were reduced relative to WT (Fig. 5B). The proportion of GC16∶0 was increased in 9H/9H liver and proportion of GC20∶0 was increased in 9H;C* spleen. No changes of GC species proportions relative to WT were found in 4L/4L and 4L/C* in visceral tissues (data not shown). The homozygous mutants, 9V/9V, 4L/4L and 9H/9H, did not show differences in GC species proportions in visceral tissues, except for GC16∶0 in 9H/9H liver. The data suggest that a greater preference of short chain GC species than long chain species with the D409V mutation in the lung. Although massive amounts of all GC species accumulated in 9H;C* liver, the proportions of the species were the same as WT, showing a lack of GC species preference for degradation by the D409H GCase.

About 80% of total GCs in the WT brain are GC18∶0 (Fig. 5C). The proportion of GC18∶0 was significantly increased only in the 4L;C* brains using the one-way Tukey’s HSD test. In 9H;C* brains, the GC18∶0 proportion was reduced, whereas and proportions of long chain GCs, GC24∶0 and GC24∶1, were increased; This was not observed in other mutants. No changes in the proportion of GC18∶0 was found in other *Gba1* mutant brains. The data indicate an association of brain GC18∶0 with the V394L mutation. The long chain species found in 9H;C* brain could derive from macrophage infiltration as suggested for Gaucher disease type 3 brains [10], [25].

Glucosylsphingosine concentrations were greatly increased in 4L;C* brains and mildly elevated in 9H;C* brains. These levels were significantly different from WT and other mutants when analyzed with Tukey’s HSD test (Fig. 5D). In visceral tissues, glucosylsphingosine was significantly increased in liver, lung, and spleen of 9V/null and 4L;C* spleen, but no differences were found in the other mutants. These imply tissue specific defects in glucosylsphingosine degradation that are associated with D409V in viscera and V394L and D409H in the brain.

### Age-dependent Change of GC Species and Glucosylsphingosine

**Figure 6:**
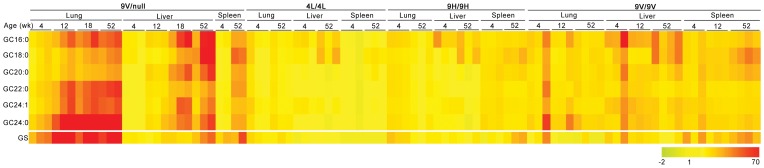
Heatmap of GC species in *Gba1* mutant mice. The fold changes of GC species and glucosylsphingosine levels in individual mutant mice were age-grouped for each organ according to genotype using Gene Spring 11.5.1. The fold change was obtained for each condition relative to the average value of the age-matched WT control. The color gradients represent level of fold change. Yellow indicates that the GC species levels in the mutant were at WT levels and the fold change was 1. The gradient from yellow to red indicates GC species levels in the mutants were above WT levels and fold change were >1, and yellow to green represents <1. 9V/null mice showed age-dependent increases of all GC species and glucosylsphingosine in the liver, lung, and spleen. Predominant accumulation of GC24∶0 in the lung was in 9V/null, but not in other three mutants. GC16∶0 and GC18∶0 were major species in the liver of all four mutants with a trend toward age-dependent increases. In the spleen, C18∶0 was major increased GC species in 9V/null, 9H/9H and 9V/9V, but not in 4L/4L. Age-dependent increase of glucosylsphingosine (GS) levels was detected in all three tissues in 9V/null and the spleen of 9H/9H and 9V/9V (n = 3–4 mice).

Age-dependent changes of each GC species and glucosylsphingosine were determined in visceral tissues of *Gba1* mutant mice and data are presented as a fold-change heatmap relative to age-matched WT (Fig. 6). Age-dependent increases of all GC species and glucosylsphingosine were clearly shown in 9V/null liver, lung, and spleen. In contrast to 9H/9H, 4L/4L and 9V/9V, in 9V/null lung GC24∶0 was the predominant accumulated. GC16∶0 and GC18∶0 were the major species in the livers from all four mutants with a trend of age-dependent increases. In the spleen, GC18∶0, a low abundant GC species, showed the greatest increase in 9V/null, 9H/9H and 9V/9V mice, but not in 4L/4L. Age-dependent increases of glucosylsphingosine levels were detected in all three tissues from 9V/null and in the spleen of 9H/9H and 9V/9V. These results revealed tissue specific, age-dependent accumulations of GC species that relate to the particular *Gba1* mutant. The heat map provides a global view of those substrates levels in relation to the tissues, age and genotypes.

**Figure 7:**
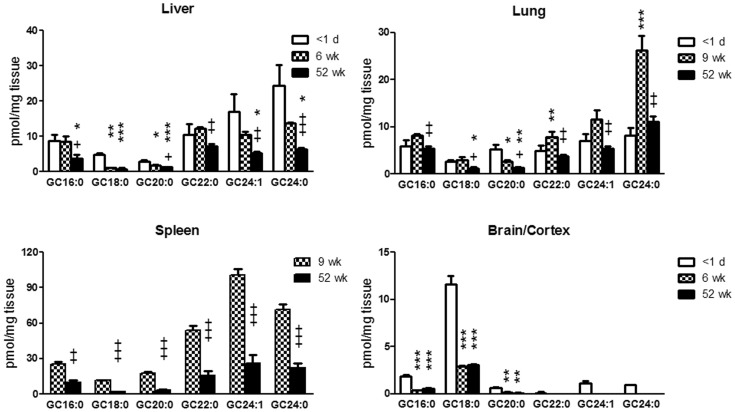
Age-dependent changes of GC species levels in WT mouse tissues. All GC species in WT mice showed age-dependent decrease with age (from <1 d, 6–9 wks to 52 wks) in visceral tissue and brain (<1 d and 6 wks) or cortex (52 wks) extracts. Selected GC species in newborn brain (<1 d) had ∼ 3-fold higher levels than that in mature mice brain. Each GC species level at 52 wks was analyzed relative to 1 d (*) or 6–9 wks (+) by Student’s t-test. * or +, p<0.05, ** or ++, p<0.01, *** or +++, p<0.0001. The results represent the mean±S.E. (n = 3–6 mice).

GC species concentrations in WT mice were compared throughout development (1 day to 52 wks) and in different tissues (liver, lung, spleen and brain). Substantially higher concentrations of GC species were detected in about 1-day old liver and brain relative to that in 6, 9, and 52 wks WT mice (Fig. 7). Although the levels of the GC species changed with age, the proportions of each GC species, relative to total GC, were the same at 6 and 52 wks (Fig. 5). There were clear developmental changes in the tissue contents of each of the GC species from these normal mice with progressive decreases in each of the various species with age.

## Discussion

Great interest in understanding phenotypic variation in single gene disorders has focused on genotype/phenotype correlations with little attention paid to the effects of missense mutations on specific metabolic derangements in selected tissues *in vivo*. Here, several missense mutations in *Gba1*, either isolated or in combination with selective saposin C deficiency, were analyzed to assess the tissue and/or mutation specific effects and their potential implications for phenotypic variation [1]. Saposin C has two effects related to GCase: 1) *in vivo* proteolytic protective effect for WT and mutant GCases [19], [21], [23], and 2) catalytic optimization effects to enhance GCase activity [26]. The saposin C deficient (WT;C*) mouse, which does not accumulate GC or glucosylsphingosine within the time frame of these experiments, were used to diminish the *in vivo* GCase activities of homozygous *Gba1* mutations. Central to these studies was to delineate the potential for threshold levels and substrate preferences of various GCase mutant enzymes as a basis for some of the disease effects in various tissues. Variation in the nature of GC species was evident in different tissues from WT and *Gba1* mutant mice. For example, the major GC species in WT CNS was GC18∶0 and in visceral tissues were GC16∶0, GC22∶0, and GC24∶0, but the accumulated GC species did not reflect these WT proportions in all comparable tissues of the *Gba1* mutant mice. The proportion of long chain GC24∶0 species in lung of 9V/null mice were ∼2-fold greater than those in WT lung. Also, significant increases in the GC18∶0 proportion were only found in 4L;C* brain. In addition, glucosylsphingosine accumulations in viscera were found in 9V/null, and in the brain of 4L;C* and 9H;C*, whereas these were much greater in the 4L:C* brains and less in 9H;C* brains. These results demonstrate mutation-specific quantitative differences in the accumulation of GC species and glucosylsphingosine. The homozygous mutants, 9H/9H, 9V/9V and 4L/4L, did not show significant preferential substrate accumulations, potentially reflecting the *in vivo* level of residual activity above a specific threshold.

All the mutants used in the analyses were in the C57BL/129Sv, except that 9V/null mice were in the C57BL/129Sv/FVB background. FVB background was introduced from backcrossing 9V/9V in C57BL/129Sv with *Gba1* null mice in FVB to create 9V/null. The GC and glucosylsphingosine accumulations in 9V/null mice were evident when compared to WT in C57BL/129Sv, FVB or strain-matched C57BL/129Sv/FVB (unpublished observation). Since the strain background of the 9V/null mice was different from other mutants, GC and glucosylsphingosine levels from two different strain background WT (C57BL/129Sv, FVB) mice were included in the analyses. The analyses demonstrated that substrate preferential accumulation is associated with the mutation in the genetic background matched mutants, 9H;C* and 4L;C* models, as well as in the mixed background 9V/null. However, elucidation of the effect of genetic background on Gaucher disease GC and glucosylsphingosine levels would require a systematic investigation against pure inbred mouse strains, which is beyond the scope of this study. Certainly this should be taken into consideration in human populations, which are never purely inbred.

Specific threshold levels of GCase were evident in *Gba1* mutant mouse tissues. In the 9V/null or 9H;C*, 9V;C* and 4L;C* mice, the mutant GCase *in vitro* activity levels were reduced by a null heteroallele or in the absence of saposin C (C*). In WT;C* mice, GC and glucosylsphingosine levels are not significantly altered in the visceral tissue and brain, but ∼50% reductions of WT or mutant GCase proteins and *in vtiro* activities are prominent due, in part, to increased GCase proteolysis in the lysosome [19], [21], [23]. The various *Gba1* homozygotes showed small to moderate increases in GC species in various tissues and these were relatively uniform across the GC species and not significantly different between each homozygotes, except GC16∶0 in 9H/9H liver, which likely due to residual activity above the threshold. In the 9V;C* and 4L;C* mice, lower GCase *in vitro* activities were present in visceral tissue and/or brain, and these mice accumulated much greater amounts of substrates compared to their respective *Gba1* homozygous mutants. In comparison to *Gba1* homozygotes, the 9V/null and 9H;C* would be expected to have ∼50% GCase (*in vivo*) of that present in 9V/9V or 9H/9H mice, respectively. This difference in GCase activity, although not clearly evident by *in vitro* assays, resulted in major increases in substrate accumulations, particularly in visceral tissues. In contrast, lower GCase in 0S;C* mice did not cause significant substrate accumulation due to the very young age of these mice (∼1 day). Thus, threshold levels of GCase are reflected by substrate accumulation and affected by mutation and tissues.

Such GCase threshold levels resulting from specific mutations also affected glucosylsphingosine degradation. Glucosylsphingosine is the deacylated form of GC. In viscera, only the 9V/null lung showed very large increases in this substrate, although the 9V;C* lungs had increased glucosylsphingosine at one day of age, whereas the 9H;C* at 52 wks showed little difference from WT. This reflects the differential effects of a single amino acid substitution at the same residue. In brain, significant accumulations of glucosylsphingosine were found in the 4L;C* mice and these levels were ∼2-fold greater than the excesses of this substrate in the 9H;C* mice. The former were 6 wks and the latter were 52 wks. None of the 9V variants showed increased glucosylsphingosine in the brain. The glucosylsphingosine accumulations in the ∼1 day old 9V;C*, or 0S;C* mice were not significantly elevated. No significant accumulation of gluosylsphingosine found in any of the *Gba1* homozygotes. These findings demonstrate that glucosylsphingosine accumulations related to the genotype and the *in vivo* residual enzyme level in specific tissues. The study also suggests that measuring substrate levels could be an alternative way to estimate *in vivo* enzymatic function since subtle changes of the enzyme levels are not necessarily appreciated by routine *in vitro* enzymatic assays.

Preferential accumulations of GC species in each mutant and in specific tissues were striking. The 9V/null and 9V;C* had a similar profiles with longer FAAC predominating (albeit at 52 wks. vs. 1 day). The massive accumulation of GC species in the 9H;C* was more uniform across the shorter and longer FAACs in the liver, but with no preferential species dominant in the pool. In contrast, a significant increase of the longer GC24∶0 was in the 9V/null lung. The increased proportion of GC24∶0 and the reduction of shorter FAAC length GCs implies that D409V favored substrates with the short chain vs. the long chain FAACs which led to progressively greater accumulations of long chain GC. Curiously, such differential effect on GCs was not found in D409H mutant that differed from D409V by single amino acid change. In the GCase structure, the amino acid, D409, is at the top of the helix turn facing outside. Mutations in which D409 is replaced by Histidine or Valine, which changes the properties of D409 from negatively charged hydrophilic amino acid to either neutral/positive charge/hydrophilic or hydrophobic amino acid. This change affects the crystal structure of GCase leading to altered catalytic function of GCase towards its substrates, as reflected by D409H having a 2-fold increased IC50 for deoxynojirimycin, an active site-directed inhibitor, compared to that for D409V [4]. The V394L mutation had greater effects on brain GC18∶0 and glucosylsphingosine degradation than mice with the D409H or D409V mutations. Again, reflecting a mutation dependent substrate preference that could contribute to phenotypic variations. The expected result in the null/null mice, if they had survived, would be a progressive accumulation of GC in large amounts in tissues, but the GC species would be expected to be in the same proportions as found in the corresponding WT tissues.

The GC species profiles and glucosylsphingosine accumulation in mutant mice were also tissue specific. In lungs, similar patterns for the various genotypes were found, but the total amounts of GC species were much greater in the 9V variants compared to 9H variants. However, the preferential accumulation of longer GC species was evident in all visceral tissues of the 9V variants. The brains showed more uniform and smaller accumulations of GC species with GC18∶0 predominating and with significant glucosylsphingosine accumulation in the brain of 4L variant.

Curiously, no direct relationship was found between the degrees of GC and glucosylsphingosine accumulations in the various tissues. If the substrate levels were completely dependent on the residual GCase activity in all tissues, then the degrees of GC and glucosylsphingosine would have been expected to be directly correlated. For example, the 9H;C* mice has massive increases in GC in liver, but almost no increase in glucosylsphingosine. In comparison, the 4L;C* mice had similar degrees of increase in CNS GC and glucosylsphingosine, but very little in visceral tissues. This relationship was similar in the lungs from 9V/null mice with increased GCs and glucosylsphingosine. The V394L and D409H or D409V enzymes have different *in vitro* kinetic properties toward the GC and glucosylsphingosine substrates with the V394L GCase having a 3- to 5-fold decreased binding of glucosylsphingosine compared to the normal Km for GC [4]. Using synthetic substrates, the estimated catalytic rate constants of the partially purified mutant GCases were 2–3 fold greater with V394L compared with D409H or D409V [4]. However, to explain the tissue differences in the substrate accumulations based on such kinetic differences alone seem wanting and additional GCase and/or tissue specific effects will need to be invoked. Potentially additional properties could be revealed by differential processing of the various GCase proteins between species (e.g., human vs. mouse) and/or tissues. Various mutant GCases have less *in vitro* stability than the WT and several human variants have been shown to have decreased half-lives in cultured cells [4], [27], [28]. Also, the half-life of newly synthesized GCase in human fibroblast is about 60 hrs, which is slower than that in mouse fibroblasts [21], [29]. However, the overall effects of such potential differential processing of GCase in selected cell types in viscera and CNS needs elucidation to add to the additional effects of these mutation on overall GC and glucosylsphingosine fluxes.

GC has an essential role in survival [30]. Severe neurodegeneration in the conditional GC synthase knockout mice demonstrated the importance of GC and derived glycosphingolipids in the maintenance of the CNS [31], [32]. Clear developmental changes in GC species were evident in WT mice. In all tissues, greater concentrations of GC species were present at younger ages with progressive decreases in many tissues with age. This was clear in the liver from <1 day to 52 wks in all GC species, whereas in lung the reduction was greatest between 6 and 52 wks, particularly with GC24∶0. A similar trend was present in the spleen. In brain, this effect was major on the GC18∶0 species. The higher levels of GC species during early development indicate an increased demand for GC species during cell proliferation and organ maturation. Regulation of GC expression throughout development anticipates its physiological importance.

The GC species preferential distribution in the Gaucher disease mouse models mimic the findings in human patients (Table 3). Substrate and tissue specificity were implicit from these early studies of human Gaucher disease patients. GC18∶0 level is up in the brain of types 2 and 3 patients, whereas longer FAAC length GC species are increased in the spleen [7], [10]. The increased GC24∶0 in type 1 and type 3 brains was suggested coming from infiltrated macrophages lining periventricular region [15]. Long FAAC GC species were increased in type 1 Gaucher disease patients’ spleens [7], [10], whereas GC18∶0 and glucosylsphingosine are present in large amounts in neuronopathic Gaucher disease patient brains [10], [15], [17]. A correlation of particular mutations with glucosylsphingosine levels also was suggested [33]. However, the relationships of the lipid accumulations to mutations and residual enzyme activities have been elusive. The findings from Gaucher disease mouse models revealed a link between specific substrate accumulation to the selected mutation. This study is a prelude to understand the differences in substrate levels and phenotype variations caused by different mutations that may not be revealed from the residual activity.

**Table 3:** Accumulations of GC species and GS in Gaucher disease patients and mouse models.

	Human^a^			Mouse		
Tissue	GD Type	GC species^b^	GS^c^	Mutation (model)	GC species^d^	GS
Brain	Type 1	C22∶0/C24∶0	(+)	V394L (4L;C*)	C18∶0	(+++)
	Type 2	C18∶0	(++++)	D409H (9H;C*)	C18∶0	(+)
	Type 3	C18∶0	(+++)			
Spleen	Type 1	C22∶0/C24∶0	(+++)	D409H (9H;C*)	C20∶0	(-)
	Type 2	C22∶0/C24∶0	(+++)	D409V (9V/null)	np	(+)
	Type 3	nd	(+)	V394L (4L;C*)	np	(+)
Liver	Type 1	nd	(+++)	D409H (9H/9H)	C16∶0	(-)
	Type 2	nd	(+++)	D409V (9V/null)	np	(+)
	Type 3	nd	(+++)			
Lung	Type1/2/3	nd	nd	D409V (9V/null)	C24∶0	(+++)

a Summarized from [10], [15], [33], [34].

b Increased GC species level relative to unaffected control.

c (+ to ++++) indicates degree of the GS level increase. (-) indicates unaltered GS level.

d Proportion of GC species increases relative to WT indicating preferential storage.

nd, not determined.

np, no preferential storage.

GS, glucosylsphingosine.
